# Translation, cultural debugging, and validation of the Chinese version of the Sour Seven Questionnaire: a cross-sectional study

**DOI:** 10.3389/fmed.2024.1412172

**Published:** 2024-09-25

**Authors:** Shichao Zhu, Shiqing Liu, Liming Li, Huanmin Xing, Ming Xia, Guangyan Dong

**Affiliations:** ^1^Intensive Care Unit, Henan Provincial People’s Hospital, Zhengzhou, China; ^2^Henan Provincial Key Medicine Laboratory of Nursing, Zhengzhou, China; ^3^Department of Oral and Maxillofacial Surgery, The First Affiliated Hospital of Zhengzhou University, Zhengzhou, China; ^4^Department of Nursing, Henan Provincial People’s Hospital, Zhengzhou, China

**Keywords:** Chinese version, delirium, family member, nursing, Sour Seven Questionnaire

## Abstract

**Introduction:**

Intensive care unit delirium (ICUD) is an acute cerebral dysfunction accompanied by a change of level of consciousness, disorientation, and cognitive dysfunction, typically occurring over a short duration ranging from hours to days and resulting from underlying medical causes. Family members may sometimes detect changes in consciousness earlier than medical staff. The Sour Seven Questionnaire is a tool to assist family members in screening for delirium, but there is currently no Chinese version. This study aimed to translate and cross-culturally debug the Sour Seven Questionnaire and test the effectiveness of the Chinese version in screening for ICUD by family members.

**Methods:**

To create the Chinese version of the questionnaire, the questionnaire was first translated and then culturally debugged through expert consultation and cognitive interviews. Patients and their family members admitted to three ICUs in a Chinese hospital were selected to test the Chinese version of the Sour Seven Questionnaire and the results were compared with those of the validated and recommended Confusion Assessment Method for the intensive care unit (CAM-ICU) assessment.

**Results:**

A total of 190 ICU patients and their families were included in this study. Results of the CAM-ICU assessment showed that 73 (38.4%) patients developed ICUD compared to the 66 (34.7%) using the Chinese version of the Sour Seven Questionnaire, which had a Cohen’s kappa coefficient of 0.853, a sensitivity of 0.863, and a specificity of 0.974. The positive predictive value was 0.954 and the negative predictive value was 0.919.

**Discussion:**

The Chinese version of the Sour Seven Questionnaire is a valid assessment tool for helping families screen for ICUD, and it is effective in identifying altered consciousness in patients even during online visits.

## 1 Introduction

ICU Delirium (ICUD) is characterized by a disturbance of attention, orientation, and awareness that develops within a short period of time, typically presenting as significant confusion or global neurocognitive impairment, with transient symptoms that may fluctuate depending on the underlying causal condition or etiology (1–3). The incidence rate of ICUD ranges from 33.1 to 80% (1–3). ICUD occurrence in patients increases the risk of hospital-acquired infections and mortality, prolongs hospital stay, and generates higher hospitalization costs (4). ICUD could lead to an increase in hospital costs of $4,654 per patient (5). The cost of delirium treatment in the United States increased from $16 billion (2004) to $182 billion (2021) (6, 7). This shows that ICUD poses a significant challenge to patient health and healthcare resources.

ICU nurses are the healthcare professionals who spend the most time with critically ill patients, being the best personnel to assess ICUD. Guidelines (2, 8) and consensus (1, 9) recommend the CAM-ICU or ICDSC as valid screening tools for ICUD. However, Lange et al. (10) found that 53.1% of ICU nurses had never received education on delirium, 16.4% routinely assessed delirium, 35.8% occasionally assessed delirium, and they rarely used validated scales. A survey from China noted that only 10.81% of ICU nurses were familiar with delirium, 11.49% routinely used a delirium assessment tool to screen for it, and 37.17% assessed patients based on their own experience (11). Family members usually know the patient best and may notice subtle changes in the patient’s cognition and behavior earlier than nurses (12). Therefore, family members’ involvement in assessing ICUD can assist in early diagnosis and provide valuable time for early intervention. When assessment tools are not used, delirium is often missed diagnosis, with nurses identifying only 35% of delirium cases (13), resulting in many patients with ICUD not being identified. Several family versions of assessment tools have been developed to fully utilize the role of families in delirium assessment (14). However, only the Family Confusion Assessment Method (FAM-CAM) (12) and Sour Seven Questionnaire (15) have been validated for use in ICUD assessment, with the latter proving superior in assessing delirium (12).

Given that patient benefits of conducting an ICUD assessment outweighs the potential risks, family members may be more likely to recognize delirium symptoms than nurses who are unfamiliar with the patient (8, 14). However, there is no Chinese version of this tool for patients’ families. Therefore, we aim to culturally debug and clinically apply the Sour Seven Questionnaire to explore its effectiveness in predicting ICUD and lay the foundation for further in-depth research.

## 2 Materials and methods

### 2.1 Location

This study was conducted in three ICUs (medical, surgical, and comprehensive) of 30 beds each, of a 4,550-bed hospital in China. This study is reported according to the Standards for the Reporting of Diagnostic Accuracy Studies (STARD) (16). This study was approved by the Ethics Committee of Henan provincial people’s hospital (Approval no. 2021-94). This study was registered in the National Information Platform for Universal Health Coverage (NPUHC) medical research registry case information system,^1^ (MR-41-22-022684). In this study all participants provided written informed consent prior to recruitment. The accession numbers of data have not yet been obtained at the time of submission, but we can provided the data to reviewer, if it is needed.

### 2.2 Participants

In this study, a convenience sampling method was used to collect data. The participants of this study were patients admitted to the three ICUs between July 2022 and May 2023. The inclusion criteria were: (1) age ≥ 18 years; (2) ICU stay ≥48 h; (3) RASS score ≥ −2; (4) able to communicate; (5) and agreeing to voluntary participation in this study. Patients were excluded if they presented (1) a history of mental illness and (2) diagnosis of delirium before ICU admission.

In this study, family members were defined as those who lived with the patient or had contact with the patient at least once a month and were familiar with the patient’s habits and personality. The inclusion criteria for family members were: (1) age ≥ 18 years old; (2) ability to use a smartphone; (3) ability to participate in visits on time and throughout the entire study period; (4) ability to communicate; and (5) agreeing to voluntary participation in this study.

The sample size assessment was based on sensitivity as the main indicator (17). The predicted sensitivity of the Chinese version of the Sour Seven Questionnaire was 80%, 1-β = 0.9, and ICUD incidence 33.1%. Therefore, the required sample size was 186 cases.


n=Z^2×P1−P/β^2N=n/Prevalence

$$\left(Z=1.96,\;P=0.80,\;\beta =0.1,\;\text{Prevalence}=0.331\right)$$

### 2.3 Status of the target scale

The Sour Seven Questionnaire (SSQ) was developed by Shulman et al. (18) and contains seven entries. While it has a total score of 18, patients are at high risk for delirium when they score ≥ 4, and delirium is occurring when they score ≥ 9. The sensitivity of the validation in Canadian ICU patients was 0.64 and specificity 0.85 (18). Furthermore, it has been noted that the SSQ has a positive and negative predictive value of 89.5 and 90%, respectively (14).

### 2.4 Translation and cross-cultural adaptation process

We obtained the SSQ authorization and adaptation in accordance with ISPOR guidelines (19). Two ICU nurses (Both translators are native Chinese speakers. They hold a master’s degree in nursing in Ireland and have significant experience working in intensive care units (ICUs). One translator has 8 years of ICU experience, while the other has 6 years. Their involvement in ICU delirium research is extensive, having participated in several projects and co-authored papers with the research team) independently completed the translation. The research team leader reviewed the two translations and original scale, discussed inconsistencies, and coordinated revisions to develop a comprehensive Chinese version. This version was presented as accurately as possible in easy-to-understand language based on the meaning of the original scale items. Two master’s students who had not read the English version of the SSQ independently reverse translated the Chinese version. The research team leader reviewed both translations and the original scale. The final translation was discussed and approved by the research team core members.

Four ICU medical specialists, three ICU nursing specialists, and the head nurse of the Department of Psychological Medicine were requested to evaluate the content of the entries in the Chinese version of the Sour Seven Questionnaire (CVSSQ). The appropriateness of each scale entry was evaluated using a 5-point Likert scale (1 = “not at all appropriate” and 5 = “very appropriate”). For entries with scores of 1–3, the experts were requested to provide modification suggestions, while the research team discussed and developed the CVSSQ. The modified scale was sent back to the eight experts for a second round of consultation until consensus was reached.

#### 2.4.1 Permission to reuse and copyright

Permission must be obtained for use of copyrighted material from other sources (including the web). Please note that it is compulsory to follow figure instructions.

### 2.5 Pre-survey

A total of 30 pairs of family members and patients who met the inclusion and exclusion criteria were recruited to use the CVSSQ in the pretest phase. After the family members assessed the patients, they were interviewed by the researcher to collect their opinions on the tool. Modifications were made based on their opinions.

### 2.6 Delirium assessment

The CAM-ICU has a good diagnostic ability for delirium and can be conducted rapidly (8). Trained nurses used it as a diagnostic tool for delirium.

### 2.7 Data collection

#### 2.7.1 Nurses grouping and training

As each ICU contained four nursing groups, we recruited one nurse in each group (12 nurses in total) responsible for assisting family members to conduct online visits (RT1). Simultaneously, we recruited another nurse in each nursing group (12 nurses in total) who were proficient in using the CAM-ICU to be responsible for assessing delirium (RT2). After completing their training, the RT2 nurses first underwent a theoretical examination. Once they passed the theoretical examination, the nurses were required to evaluate standardized patients using the CAM-ICU to demonstrate their proficiency in using the CAM-ICU correctly. Upon passing these assessments, the RT2 nurses were eligible to participate in the study.

#### 2.7.2 Family-administered delirium assessments

**Figure 1:**
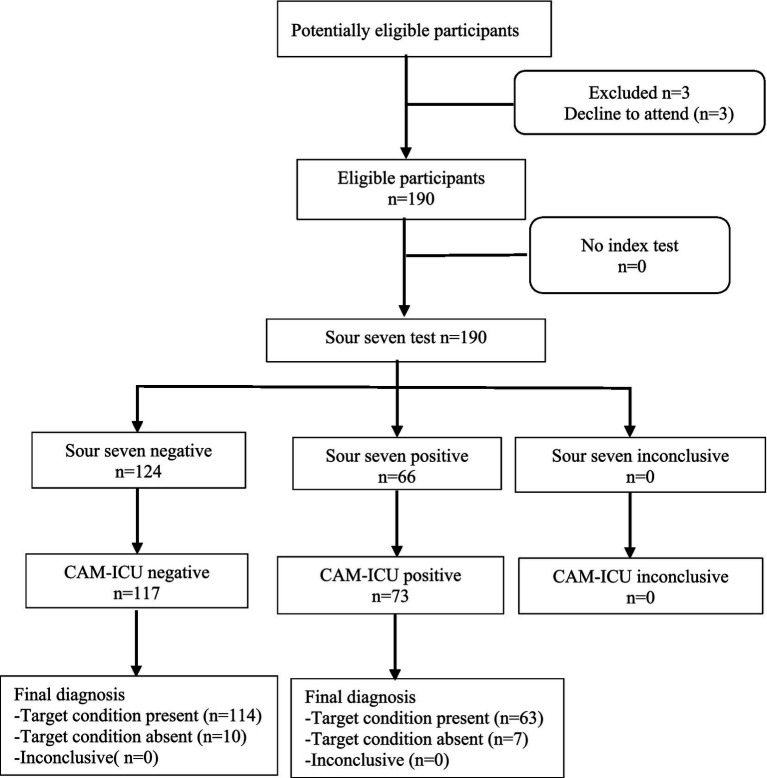
Flow diagram of participants.

When patients who met the inclusion and exclusion criteria were admitted to the ICU, the nurse from RT1 was responsible for obtaining informed consent from the family and the patient. This nurse informed the family that only one member could participate in this study and gave a paper copy of the CVSSQ to the family before each online visit. Because the Sour Seven Questionnaire is a novel, brief, easy-to-use clinical tool, we did not have training for the families (20). The nurses from RT1 assisted the family with a 20-min online visit daily from 10:00 to 11:00 and 21:00 to 22:00 every day. During the visit, the family assessed the patient using the CVSSQ, and the results were maintained by the RT1. In this study, a CVSSQ score of ≥9 points is the screening criterion for ICUD positivity (20). When the family used the CVSSQ to assess whether the patient had an ICUD, a score of <9 was classified as ICUD-negative, while a score of 9 or greater was classified as ICUD-positive. When the family is unable to determine whether the patient’s performance aligns with what is described on the CVSSQ, we consider the assessment to be “inconclusive” (see Figure 1).

#### 2.7.3 Clinical delirium assessments

One hour after the family’s visit, the nurses from RT2 assessed the patient for delirium using the CAM-ICU. The results of CAM-ICU were maintained by the RT2. The RT1 and RT2 were unaware of each other’s delirium assessment results.

#### 2.7.4 Stopping the assessment

The assessment was stopped when the patient was transferred out of the ICU, or diagnosed with delirium by the CAM-ICU or CVSSQ. For example, when a patient has a CAM-ICU score of 4 but a CVSSQ score of 3, the assessment can be stopped. At this point, the patient is considered to have been assessed with a positive CAM-ICU and a negative CVSSQ result.

#### 2.7.5 Quality control

Each week, one investigator who is skilled in the use of the CAM-ICU reassessed the patient using the CAM-ICU within 1 h of the end of the assessment for RT2. The assessment of RT2 was considered valid if the results were consistent; if they differed, another investigator who was skilled in the use of the CAM-ICU performed the assessment.

### 2.8 Statistical analysis

Statistical analysis was performed using IBM SPSS Statistics for Windows, Version 26.0 (IBM Corp). Data that did not follow a normal distribution are reported as medians [interquartile range] and count data are expressed as percentages. Validity analyses were conducted using the item-level content validity index (I-CVI), scale-level content validity index (S-CVI), and diagnostic validity. The reliability analysis was validated using the Cohen’s kappa coefficient and inter-rater reliability. Sensitivity, specificity and positive and negative predictive values for the CVSSQ were calculated using crosstabs. The area under the receiver operating characteristic curve (AUROC) AUROC was used to analyze the ability of delirium detection for the CVSSQ. When mapping the AUROC, the screening results for delirium were converted into binary variable: CAM-ICU-positive (≥4 points), CAM-ICU-negative (<4 points); and CVSSQ-positive (≥9 points), CVSSQ-negative (<9 points). The CAM-ICU results were used as the status variable. Differences were considered statistically significant at *p* < 0.05.

## 3 Results

This study included 190 patients, of whom 73 (38.4%) developed ICUD (see Figure 1). Patients’ median age was 55 [47, 63] years, 99 (52.1%) were male, and 108 (56.8%) were medical patients while 82 (43.2%) were surgical (see Table 1). The family members’ age averaged at 51 [42, 59] years old, and 101 (53.2%) were male (see Table 2).

**Table 1:** General information of ICU patients.

Items	Delirium *N* = 73	Non-delirium *N* = 117	Value	*P*
Gender (female), *n* (%)	28 (38.6)	63 (53.8)	190.000^a^	<0.01
Age, year (Age, median [IQR])	62 [52, 68]	53 [45, 60]	4.621^b^	<0.01
Marital status, *n* (%)			8.577^c^	0.02
Single	0	4 (3.4)		
Married	65 (89.0)	107 (91.5)		
Divorced	2 (2.8)	5 (4.3)		
Widowed	6 (8.2)	1 (0.8)		
Category of disease			9.988^a^	<0.01
Medical patients, *n* (%)	31 (42.5)	77 (65.8)		
Surgical patient, *n* (%)	42 (57.5)	40 (34.2)		

^aChi-square test. bMann–Whitney U test. cFisher test.^

**Table 2:** General information of family members.

Items	Delirium *N* = 73	Non-delirium *N* = 117	Value	*p*-value
Gender (female), *n* (%)	40 (54.8)	49 (41.9)	3.011^a^	0.10
Age, year (Age, median [IQR])	53 [42, 59]	50 [41, 57]	1.482^b^	0.14
Relationship to ICU patient, *n* (%)			8.072^c^	0.03
Spouse	48 (65.7)	92 (78.6)		
Child	21 (28.8)	16 (13.7)		
Parents	1 (1.4)	6 (5.1)		
Others	3 (4.1)	3 (2.6)		
Education attainment			5.844^a^	0.12
Primary school	19 (26.0)	18 (15.4)		
Junior high school	27 (37.0)	36 (30.8)		
Senior high school	17 (23.3)	39 (33.3)		
Bachelor degree or above	10 (13.7)	24 (20.5)		
Job style, *n* (%)			2.564^a^	0.64
Medical staff	6 (8.2)	5 (4.3)		
Worker	18 (24.7)	27 (23.1)		
Peasants	26 (35.6)	37 (31.6)		
Office worker	9 (12.3)	19 (16.2)		
Other occupation	14 (19.2)	29 (24.8)		
Marital status, *n* (%)			2.940^c^	0.15
Single	0	3 (2.6)		
Married	72 (98.6)	114 (97.4)		
Divorced	0	0		
Widowed	1 (1.4)	0		
Frequency of contact with ICU patient, *n* (%)			0.468^a^	0.494
Direct contact at least once a month	7 (9.6)	8 (6.8)		
Living together	66 (90.4)	109 (83.2)		

^aChi-square test. bMann–Whitney U test. cFisher test.^

### 3.1 Validity

#### 3.1.1 Content validity

After being scored by the eight experts, the CVSSQ used in this study had an I-CVI and S-CVI score of one for each entry.

#### 3.1.2 Diagnostic validity

**Figure 2:**
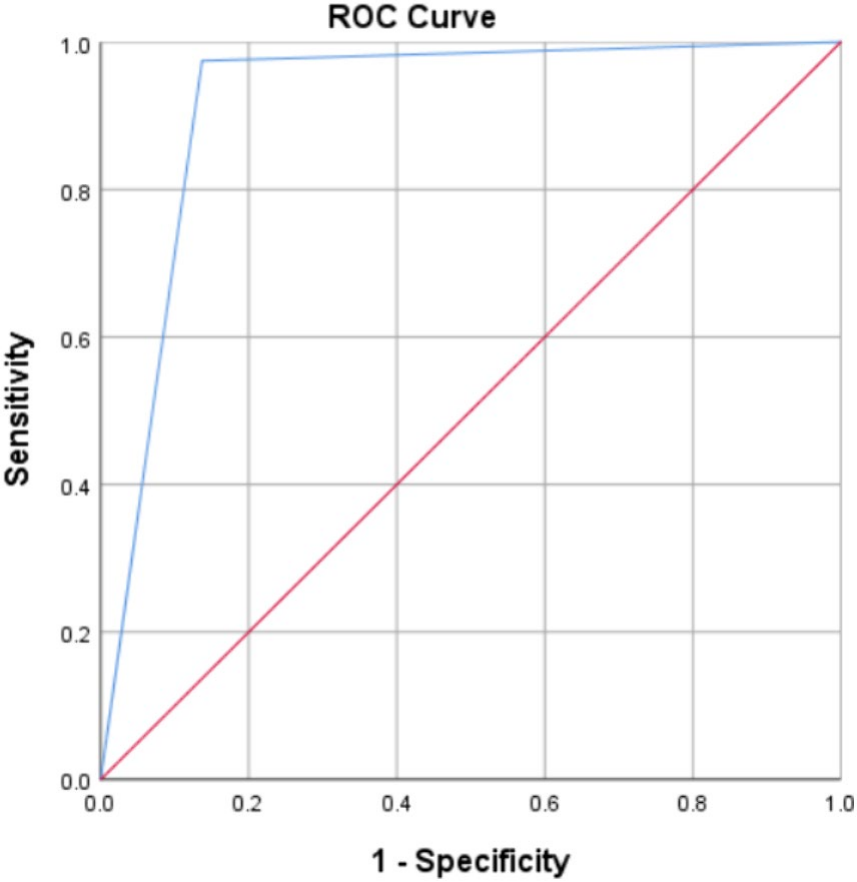
ROC curve of the Chinese version of the Sour Seven Questionnaire to predict ICU delirium.

Cohen’s kappa coefficient of the CVSSQ and CAM-ICU diagnostic results in this study was 0.853 (95% CI 0.777–0.929, *p* < 0.01). The sensitivity, specificity, positive, and negative predictive values of the CVSSQ were 0.863, 0.974, 0.954, and 0.919, respectively (see Table 3). The AUROC for the CVSSQ was 0.919 (95% CI 0.869–0.969, *p* < 0.01; Figure 2).

**Table 3:** Diagnostic validity of the Chinese version of the Sour Seven Questionnaire.

		CAM-ICU		
		Positive	Negative	Total
CVSSQ	Positive	63	3	66
	Negative	10	114	124
	Total	73	117	190

CAM-ICU, Confusion assessment method of intensive care unit; CVSSQ, The Chinese version of the Sour Seven Questionnaire.

### 3.2 Reliability confidence

Cohen’s kappa coefficient between the CVSSQ and CAM-ICU assessment results was 0.853 (*p* < 0.01).

## 4 Discussion

We used the CAM-ICU as a diagnostic criterion because it is a tool for assessing delirium in the ICU that is widely used in clinical practice and has been co-recommended by multiple guidelines (1, 2, 8). In this study, the CVSSQ had a sensitivity and specificity of 0.863 and 0.974, respectively, while Moss (18) study showed a sensitivity and specificity of 0.64 and 0.85, respectively. This may be related to the difference in the frequency of assessment between the two studies. In Moss (18) study, family members conducted only one delirium assessment, whereas in this study, family members conducted two in accordance with the nurses’ assessments in order to maximize the timely identification of ICUD. Another possible reason for the difference in sensitivity between the two studies may be the fluctuating and rapid nature of delirium presentation (8). Additionally, patient sedation scores were not reported in Moss (18) study. The PADIS guidelines state that the depth of a patient’s sedation may lead assessors to make incorrect judgments: the deeper the patient’s sedation, the greater the chance of being misjudged (8). We believe that increasing the frequency of family members assessing the patient to be close to the frequency and duration of nurses’ assessments better reflects the true effect of family members using the CVSSQ to assess ICUD. Therefore, we consider this to be one of this study’s innovations.

The study period coincided with the COVID-19 pandemic; therefore, new cases of coronavirus-related pneumonia were admitted. To avoid aggravating the condition of critically ill patients in the ICU, on-site patient visits were not permitted, and many hospitals used online or telephonic visits to meet the emotional needs of patients and their families (21, 22). Compared to telephonic visits, online visits (via video) can calm patients’ emotions through real-time images, reduce the incidence of ICUD, and increase communication between patients and their families (23). Furthermore, online visits reduce the visiting costs and removes traveling barriers (23). In our study, we found that during online visits, family members could assist in diagnosing ICUD by observing the patient’s movements. This, to a certain extent, improves the chances of early diagnosis. Online visits not only reduce the risk of infection and difficulty for family members to accompany the patient, but it is also more convenient. While online visitation offers certain benefits, it also presents limitations. One study found a 74% agreement between online and in-person diagnoses and a 79.8% concordance in treatment plans (24). However, the lack of direct interaction with the patient may hinder the accuracy of screening tools like the CVSSQ. This aspect requires further investigation in future studies.

Furthermore, we analyzed whether general patient and family information affected delirium assessment using the CVSSQ. In the patient data analysis, there were differences between patients in the delirium group and those in the non-delirium group in terms of age, gender, marital status, and disease category (*p* < 0.05). The propensity of older age (1, 8), disease category (surgical patients) (1, 25), and marital status (26, 27) to induce ICUD has been demonstrated in several guidelines and studies. Whether gender can induce delirium is inconclusive. However, a study of diabetic patients undergoing coronary artery surgery showed that the risk of delirium was higher in men than in women (28). In a study conducted by Kotfis et al. (29) it was also demonstrated that the risk of delirium was higher in male critically ill patients than in females. Peckham et al. (30) concluded that males have a higher likelihood of bacterial, viral, and other infections; that females have a stronger humoral immune response than males; and that males show an age-related tendency for a decline in B-cells and accelerated immune senescence. The above reasons may contribute to the fact that male are more prone to inflammatory responses. Inflammatory response is one of the important factors that induce ICUD (31, 32).

Family members are the primary users of the CVSSQ, and their perceptions can influence the results of the CVSSQ assessment. Therefore, we recommend more focus on the analysis of family information. The difference in the category of family members in this study was statistically significant (*p* = 0.03), which may be related to spouses who lived together for a longer period having a better understanding of each other. Although the differences in education and occupation between the two groups of family members were not statistically significant (*p* > 0.05), they still suggest that the CVSSQ can be applied to family members with different education levels and professions, which has been less addressed in previous studies.

In this study, we excluded patients with RASS scores <−2. The PADIS guideline states that sedation level may influence the assessment of delirium, noting that many patients with a RASS score of-3 are considered “unassessable” and that the rate of positive delirium is significantly higher when patients have a RASS of-2 (as opposed to a RASS of −1 to 0) (8). Because delirium can manifest as a reduced level of arousal, families typically lack the medical knowledge to easily classify altered consciousness due to sedation as ICUD, thus making the CVSSQ diagnosis less effective (8). Chinese experts believe that “the eCASH concept” is effective in improving patients’ comfort and reducing the incidence of ICUD (9). The eCASH concept recommends that light sedation should leave the patient in a state of calmness, comfort, and cooperation (33). Ideally, the patient should be awake to maintain eye contact, interact with caregivers and family members, and participate in physical and/or occupational therapy while being allowed to drift off to sleep when undisturbed (33). This state is broadly equivalent to a Richmond Agitation Sedation Scale (RASS) score of −1 to 0 (33).

The occurrence of ICU delirium increases the risk of hospital-acquired infections and mortality in critically ill patients, prolongs hospital stays, and leads to higher hospitalization costs (4). However, public awareness of delirium lags far behind other important public health issues (32). The prevalence of ICUD in this study was 38.4%, indicating a high prevalence of ICUD in critically ill patients and reflecting the significant use of healthcare resources by ICUD. The PADIS guideline states that early detection of ICUD is essential to expedite clinical assessment and intervention, and ICU nurses are always with the patient, making them the best people to detect ICUD (8). However, several studies have shown that the ability of some ICU nurses to assess delirium does not meet clinical needs due to a lack of knowledge or inadequate training This has led to a large number of patients with ICUD being underdiagnosed in the clinic (34, 35). As the study progressed, the researchers found that family members were able to identify patients with ICU delirium earlier than healthcare professionals; that is, family members were able to recognize patients with ICU delirium earlier (14). This may be related to the fact that family members are more familiar with the patient’s habits than healthcare professionals. We translated the SSQ into Chinese and applied it to ICU patients, finding that family members can conduct delirium screening alongside healthcare professionals. Allowing family members to participate in the assessment of ICUD can improve the detection rate of delirium and is a tremendous help in achieving early identification of ICUD in the clinical setting (14). Rosgen et al. (14) suggested that family participation in ICUD assessment can help reduce negative emotions such as anxiety and depression in family members, but this still needs to be confirmed by further studies. In summary, family participation in ICUD detecting is important for addressing the emotional needs of patients and their families, as well as for assisting healthcare professionals in achieving the early identification of delirium.

In this study, we translated the Sour Seven Questionnaire into Chinese and assessed its clinical effectiveness. While our findings suggest that the CVSSQ can help families identify early ICUD, the study was limited to a single center and relied on online visits. With the increasing return to in-person assessments in hospitals, we plan to expand our research into a multicenter study. This study will involve ICUs from various regional hospitals in China and will focus on how different demographics and the mode of consultation influence the effectiveness of delirium detection.

## 5 Limitations

This study has four limitations. (1) The Diagnostic and Statistical Manual of Mental Disorders (DSM) was not used as a diagnostic criterion for ICUD which may have led to some biased results. However, using the CAM-ICU for delirium assessment is more in line with clinical practice. (2) Because we had previously used the ICUD risk prediction model to assess delirium risk in critically ill patients, we did not validate that a CVSSQ score of ≥4 was the high-risk cut-off point for ICUD; however, we validated that a patient scoring ≥9 could be diagnosed with delirium. (3) The absence of standardized training for families on the Chinese version of the Sour Seven Questionnaire (CVSQQ) in this study may have introduced variability in how families interpreted the questionnaire. This inconsistency could have impacted the accuracy of the assessment results. (4) Although we confirmed the absence of psychiatric abnormalities in patients by reviewing their medical records upon ICU admission, the lack of standardized baseline mental health and cognitive assessments remains a potential limitation. This absence of objective evaluation could influence the accuracy of subsequent assessments.

## 6 Conclusion

This study demonstrated that the CVSSQ provides a valid assessment of ICUD. It is easy to use and convenient to operate, while potentially playing a significant role in the early identification of ICUD. For an efficient assessment, it is important to promote cooperation between nurses and their families.

## Data Availability

The raw data supporting the conclusions of this article will be made available by the authors, without undue reservation.

## Footnotes

^1^ https://www.medicalresearch.org.cn
